# Biological maturity but not relative age biases exist in female international youth soccer players relative to the general population

**DOI:** 10.5114/biolsport.2025.144411

**Published:** 2024-11-05

**Authors:** Liam Sweeney, Tommy R Lundberg, Cian Sweeney, Jack Hickey, Áine MacNamara

**Affiliations:** 1 Department of Sport Science and Nutrition, Faculty of Science and Engineering, Maynooth University, Kildare, Ireland; 2 Division of Clinical Physiology, Department of Laboratory Medicine, ANA Futura, Karolinska Institutet, 14152 Huddinge, Sweden; 3 Unit of Clinical Physiology, Karolinska University Hospital, Stockholm, Sweden; 4 School of Science and Technology, Nottingham Trent University, Nottingham, United Kingdom; 5 School of Health and Human Performance, Faculty of Science and Health, Dublin City University, Dublin 9, Ireland

**Keywords:** Talent Identification, Talent Development, Female Sport, Youth Soccer, Relative Age Effect

## Abstract

This study investigated the extent to which biological maturity and relative age biases existed and varied with chronological age in female international youth soccer players relative to the general population. A total of 113 players (52 under-15 (U15), 32 under-16 (U16) and 29 under-17 (U17)) selected by the Football Association of Ireland participated in this study. All players were assessed for height, body weight and relative age. Biological maturity status and timing were assessed in U15 and U16 players only. Relative to population norms, the results showed a significant but small bias in favour of more biologically mature players (P < 0.001, d = 0.39) that increased with age from U15 (P = 0.007, d = 0.36) to U16 (P = 0.009, d = 0.44). U16 players had achieved a significantly higher percentage of their predicted adult height than U15 players (T = 7.4, P < 0.001). However, there were no relative age biases at any age group nor across the total sample (P > 0.05). There was no significant difference in height, weight or relative age of the players between the three age groups and no significant difference between the U15 and U16 age groups in terms of predicted adult height or biological to chronological age offset. This study demonstrates that biological maturity but not relative age biases exist in female international youth football relative to the general population, with notable differences in pattern and magnitude compared to those previously observed in male international youth football.

## INTRODUCTION

Identifying and nurturing youth players capable of reaching the elite senior standard of competition is of primary importance to National Football Associations and their respective football clubs. Consequently, many Football Associations worldwide have implemented talent development programmes at the local, regional, national, and international level [1, 2], which, in some contexts, begin identifying athletes during mid-late childhood at the club level [3], and from mid-adolescence at the international level [4]. Although talent identification typically happens at an early stage of the pathway, talent development is well established as a typically complex, dynamic, and non-linear process, with a range of biopsychosocial variables having the potential to impact a young player’s progression [5]. In this regard, there is substantial evidence that successful junior and successful senior athletes are largely two disparate populations [6] and that those selected early into formalised talent development programmes are often those with significant early advantages relative to their peers [7].

One of the most significant early advantages in the male context is advanced biological maturity [8]. Biological maturation is not synonymous with chronological age [9], with evidence that chronologically age-matched youth athletes can vary by as much as six years in status and timing [10]. In male youth athletes, advanced biological maturity provides physical advantages, such as increased body height, weight, muscular strength and power [2, 11–13]. These early advantages have typically led to a selection bias in favour of early maturing players across a multitude of football academies and junior international teams [2, 4, 7, 8].

The relationship between advanced biological maturity and player selection has been dominated by research specific to male populations [14]. Females experience different maturation processes, including increased fat deposition, breast development, pelvis enlargement, commencement of menstruation, and increases in joint laxity; all of which may be disadvantageous in sporting activities, and influence injury risk [15–18]. There is also evidence that females may experience a reduction in relative aerobic power with increasing chronological age [17, 19]. Moreover, whilst absolute muscular strength typically increases with advanced maturity in both sexes, the increase is less pronounced in females [18, 20]. Female youth footballers have also been shown to experience reductions in relative strength around Peak Height Velocity (PHV), which may further impact athletic performance [21]. Alongside these physical and physiological differences, early biological maturity has been associated with reduced selfesteem and increased drop-out from sport in females [15, 22].

Whilst variation in growth and maturation appears to have implications for long-term development in sport [7, 8], there is limited research on its relationship with selection in female populations. Of those few investigations specific to female footballers, Ginés et al. noted that whilst early maturing boys were overrepresented in Spanish football academies, this was not the case in females [23]. Preliminary findings based on coaches’ estimates of maturation suggest that late maturing females are underrepresented at the club and national levels of youth soccer in the United States of America [24]. Similarly, Martinho et al. reported that female youth players were generally more advanced in skeletal age relative to chronological age in a sample of Portuguese players [25]. Despite these few observations, there is a lack of research on biological maturity in female youth footballers [26], which limits evidence to inform practice [14]. This is concerning given the rapid increase in interest and the number of girls playing football worldwide [21].

While it has been suggested that biological maturity-related selection biases in talent development programmes are attributable to the Relative Age Effect (RAE) [27], there is now substantial evidence, at least in the male context, to demonstrate that biological maturity and relative age are unrelated [2, 4, 7, 8, 28]. The RAE is a selection bias in favour of those born earlier within their chronological age group at the expense of those born later in their age group and is now generally attributed to numerous factors mainly related to chronological age and experience [7]. The RAE has been documented in female youth sport, although findings in female youth football have been inconsistent [24, 29, 30]. Further investigation specific to the female game is therefore warranted to support the development of female athletes [14].

The aim of this study was to examine the extent to which biological maturity and relative age biases existed in female international youth footballers relative to the general population. To the best of our knowledge, this is the first empirical investigation to examine the simultaneous biases associated with both biological maturity and relative age in female international youth footballers. We focussed specifically on the under-15 (U15), under-16 (U16) and under-17 (U17) age groups, which represent the start of the international pathway for female footballers in Ireland.

## MATERIALS AND METHODS

### Research context

The Football Association of Ireland (FAI) is the National Governing Body for football in Ireland. As part of the FAI’s national/international talent development programme for girls, at the U15 age group, those players considered the highest performing nationally are selected into the FAI’s U15 National Academy. Players selected for this programme are exposed to additional training, coaching, sports science/medical support, and internal competition under the supervision of the FAI’s staff once or twice per month. The FAI National Academy for females is a one-year talent development programme designed to identify and prepare players for international-level football. At the end of the one-year programme, the players perceived by the FAI as the highest performing nationally are eligible for selection into the Ireland U16 international team. This study focussed on female players selected into the FAI U15 National Academy, the Ireland U16 international team, and the Ireland U17 international team during the 2024 international season.

### Participants, ethics and consent

A total of 113 players selected by the FAI in 2024 (n = 52 U15 national academy; n = 32 U16 international team; n = 29 U17 international team) participated in this study. Prior to participation in data collection procedures, each player and one of their respective parents/guardians provided informed assent and consent, respectively. Ethical approval was granted by the Maynooth University Research Ethics Committee (BSRESC-2024-37891).

### Anthropometric data and biological maturity

The biological maturity statuses of players were estimated using the percentage of predicted adult height by Khamis and Roche [31]. This method is based upon the presumption that those closer to their predicted adult height are more advanced in biological maturity than those further from their predicted adult height [31]. The Khamis-Roche method enables the prediction of each player’s adult height using the regression formula based upon age and sex-specific regression coefficients detailed by Khamis and Roche in their analysis of residents enrolled in the Fels Longitudinal Study [31]. The Khamis-Roche protocol requires each participant’s date of birth, body weight and height, and biological mid-parent height. The lead author measured each player’s body height to the closest 0.1 cm using a stadiometer (SECA 213, Hamburg, Germany) and body weight to the closest 0.1 kg using digital scales (SECA 807, Hamburg, Germany), while players were in socks and wearing light training t-shirts and shorts. Each player’s parents self-reported their height via an online form distributed by the FAI, which was subsequently adjusted for overestimation, as outlined by Epstein et al. [32]. In instances where a biological parent was not available to self-report their height (n = 3 [2.6% of the sample]), the mean value of the father or mother from that specific age group was used. As females typically achieve their adult height at ~16 years of age [33], only U15 and U16 players were included in biological maturity assessments (n = 84). Predicting adult height using the Khamis-Roche formula is as follows:

β0+β1 height+β2 weight+β3 mid−parent height

where β0 is the smoothed values of the intercepts, and β1, β2 and β3 are the coefficients by which height, weight and mid-parent height, respectively, are multiplied [31].

The height of each player was expressed as a percentage of their predicted adult height (hereafter, predicted adult height is referred to as ‘PAH’, and the percentage of predicted adult height is referred to a ‘%PAH’), which was used as an estimate of absolute biological maturity status at the time of observation. To express absolute biological maturity status as an index of biological age, each player’s %PAH was aligned with age and sex-specific reference standards in the United Kingdom Growth Reference Data [33], with 16.0 years used as the cut off for adult maturity. The age at which each player’s current %PAH aligned was identified as their biological age. A biological-to-chronological age offset (biological age – chronological age; hereafter referred to as ‘age offset’) was then calculated to represent the degree to which each player was advanced or delayed (where relevant) in terms of their relative biological maturity (i.e., higher positive value indicates more advanced biological maturity, lower negative value indicates more delayed biological maturity).

### Relative age

Players were categorised by relative age using their date of birth and the cut-off date for selection for their respective age group (in Ireland, the calendar year is used). The difference between birth date and competition cut-off date was divided by 365.25 (number of days in a calendar year) for each individual player and expressed as a decimal value (0.00–0.99; youngest to oldest, respectively) [7]. There were a combined total of 11 players who played up an age group (n = 4 U14 competing at U15, n = 7 U15 competing at U16); these players were considered as the youngest within their age group and subsequently had a negative relative age value.

### Data analysis

Data were analysed using SPSS V29 (IBM, New York, USA) and Jamovi v. 2.4.8.0 (Sydney, Australia). A series of one sampled means t-tests were used to examine the degree to which biological maturity and relative age biases existed by comparing the observed mean values for relative biological maturity (age offset) and relative age (decimal value) (for each chronological age group and for the total sample) against the values expected for the population. For relative biological maturity, 0.0 was considered a normative age offset value for the general population (this value indicates no biological maturity bias relative to aged-matched population norms). For relative age, the Government of Ireland Central Statistics Office [34] was used to calculate population norms. Specifically, for each age group, the number of females born in Ireland in each month of each year (2008, 2009 and 2010) was recorded and combined, and then expressed as a weighted average. The weighted average was calculated by multiplying the weight (the percentage of births from that month) by the relative age decimal value for that month (15^th^ of that month), followed by adding each month together and then dividing the total number by 100 (total number of births expressed as a percentage). This produced a population value (and a weighted decimal value) of 0.50. The observed mean value for relative age was then compared to 0.50 for each age group. Each t-test was calculated using a 95% level of confidence. Effect sizes (Cohen’s *d*) were used to examine the magnitude of any significant differences (trivial: < 0.2; small: 0.2–0.49; moderate: 0.5–0.79; large: 0.8–1.19; very large: ≥ 1.2) [35].

A one-way between-measures ANOVA was used to analyse differences between age groups in height, weight, and relative age of players. Unpaired t-tests were used to compare the U15 and U16 age groups in terms of PAH, %PAH and the difference between age offset. The reason that we did not include the U17 age group in this analysis of maturity status and timing is that girls are essentially fully mature at age 16 [33] and thus, we used age 16 as the adult maturity level in the equation described earlier. Analysing maturity status and timing in this age group would, therefore, yield unreliable results.

## RESULTS

### Biological maturity and relative age biases relative to population means

**FIG. 1:**
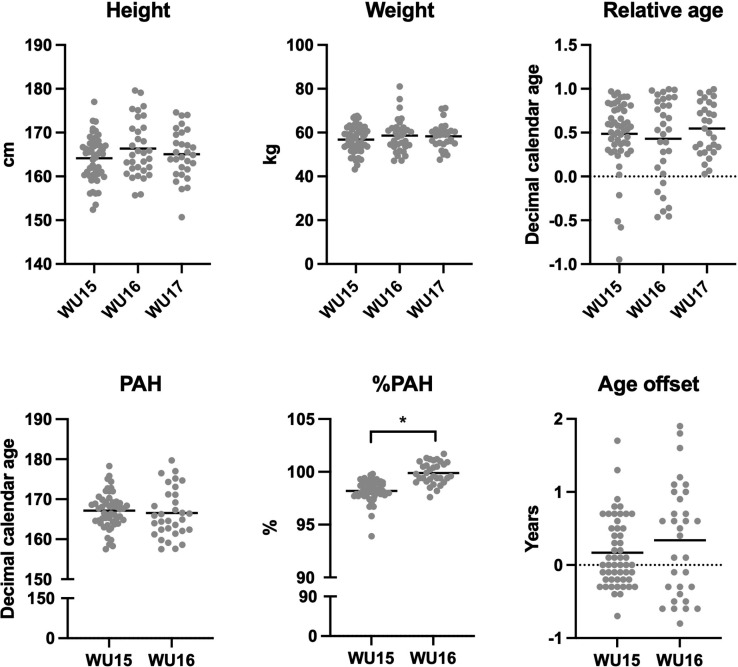
Distribution of biological maturation, relative age and physical characteristics across age groups.

Relative to population means, there was a significant bias in favour of early maturing players at U15 (P = 0.007) and U16 (P = 0.009) that increased in magnitude with age, although small in magnitude across both age groups (*d* = 0.36–0.44) (Table 1, Figure 1). For the total sample, there was a small significant bias in favour of early maturing players (P < 0.001, *d* = 0.39). However, no relative age bias was observed for any age group or for the total sample (P > 0.05) (Table 1).

**TABLE 1:** Descriptive data (presented as Mean ± SD) for the variables of interest for each chronological age group and total sample.

Variable	U15 (n = 52)	U16 (n = 32)	U17 (n = 29)	Total
**Chronological age (years)**	14.1 ± 0.4	15.0 ± 0.5	16.1 ± 0.3	14.9 ± 0.9

**Biological age (years)**	14.3 ± 0.5	15.4 ± 0.7	–	14.7 ± 0.8

**Age offset (years) (95% CI)**	0.17 ± 0.5* (0.04–0.30)	0.34 ± 0.8* (0.06 – 0.61)	–	0.23 ± 0.6* (0.01 – 0.36)

**Relative age (95% CI)**	0.49 ± 0.4 (-0.15 – 0.07)	0.43 ± 0.5 (-0.27 – 0.08)	0.55 ± 0.3 (-0.09 – 0.13)	0.49 ± 0.4 (-0.11 – 0.03)

**Birth month**	5.8 ± 2.9	4.7 ± 3.2	5.9 ± 3.5	5.5 ± 3.1

**Body height (cm)**	164.1 ± 5.1	166.4 ± 6.4	165.1 ± 5.6	165.0 ± 5.7

**Body weight (kg)**	56.8 ± 5.9	58.6 ± 7.7	58.3 ± 5.6	57.7 ± 6.4

**Predicted adult height (cm)**	167.1 ± 4.4	166.5 ± 6.0	–	166.9 ± 5.1

**Percentage of predicted adult height (%)**	98.2 ± 1.0	99.9 ± 1.0^a^	–	98.8 ± 1.3

* Denotes a significant difference between observed value and expected value.

a Denotes a significant difference between U15 and U16 age groups

#### Anthropometrics, maturity and relative age differences between age groups

There was no difference in height, weight or relative age of the players between the three age groups (all main effects F < 1.36 and all P > 0.05, Figure 1). There was also no difference between the U15 and U16 age groups in terms of PAH or age offset. However, the U16 age group had achieved a significantly higher PAH% than the U15 age group (T = 7.4, P < 0.001, Table 1, Figure 1).

### DISCUSSION

In this study, we investigated the existence of biological maturity and relative age associated biases in female youth international footballers relative to the general population and examined how such biases varied by chronological age. In respect to biological maturity, biases existed in U15 and U16 international players relative to population norms, although small in magnitude. In contrast, there were no relative age effects at any age group, nor across the total sample.

Across the scientific literature, investigations focussed on the selection biases associated with biological maturity have been dominated by male-specific samples [4, 7, 8]. The general trend across these observations is that these biases typically occur at ~11–12 years of age in professional football academies and increase in magnitude with chronological age [7, 8]. Indeed, similar trends have been observed at the junior national and international level in Ireland and Sweden, whereby male players more advanced in biological maturity are preferentially selected with increasing chronological ages or increasing levels of competition [2, 4]. At the junior international level for males in Ireland specifically, selection biases in favour of early maturing players have been observed at very large magnitudes at U15, increasing further in magnitude with age [4]. In our sample, significant biases in favour of early maturing females existed at U15, increasing in magnitude at U16, but such biases were much smaller in magnitude than those observed across male international football [4]. Moreover, there were no significant differences in age offset between U15 and U16, suggesting that these two age groups do not differ significantly from each other in relative biological maturity, despite significant differences in absolute biological maturity (i.e., %PAH).

The differences associated with advanced biological maturity and selection between our sample and those observed in international male youth football likely has a multitude of explanations. Firstly, it is important to note that biological maturity-associated selection biases in male youth football have been largely attributed to the anthropometric, functional, and physiological adaptations conferred by advanced maturity, translating into significant advantages in terms of height, muscle mass, speed, power, strength and endurance [11, 12, 20]. In the male context, Gundersen et al. [36] show positive associations between advanced skeletal age and very-high intensity running distance (> 18.5 km · h^−1^), sprint distance, and maximal speed during match-play in U14–15 players (although the association between skeletal age and performance in football-specific endurance tests were less definitive). In contrast, females experience lower absolute gains in lean muscle mass and height relative to males [18, 20], as well as additional adaptations not experienced in males, such as menstruation, and notably, large increases in fat mass [15–18]. As outlined, these physical and physiological adaptations can be disadvantageous and do not provide the on-field physical advantages to the same extent as males.

At this point, an important factor to consider is the age of selection. At the international level in Ireland, selection begins at the U15 level for female youth, which is two years later than for males (U13). Furthermore, female youth enter puberty, on average, two years earlier than males [20] and reach their adult height ~two years earlier [33]. Indeed, in the U15 age group, mean %PAH was 98.2%, with a mean chronological age of 14.1 years. Thus, the first stages of the international pathway for girls occurs at the latter stages of puberty. Indeed, females have been shown to experience the onset of the adolescent growth spurt as young as 9.5 years in some instances, with mean ages of ~12 years at PHV [20]. As such, the investigation is limited in that we miss how maturity biases may or may not exist and change in trends at earlier ages for females. This is important given that our sample are almost entirely post PHV. Nonetheless, this is the practical reality of female international youth football, where selection typically begins at the age of 14 years. This presents an area of interest moving forward, with the need to understand the dynamics of biological maturity in female youth at earlier ages, with a focus prior to international selection.

Despite the less pronounced physical adaptations resulting from advanced maturity in girls, it should not be ignored that female players were, on average, early maturing in our sample. From a physical perspective, earlier maturing girls are typically taller and stronger in absolute terms [15, 18, 20, 21]. Thus, there are still potential physical advantages conferred by advanced biological maturity in females, particularly in terms of absolute lower body muscular strength [21, 37]. However, it should also be noted that previous investigations focussed on biological maturity and selection in female youth football [23–25] have used different methods to assess maturity (e.g., maturity offset method, radiographs for skeletal age, and simply asking coaches to estimate), which all differ to our approach (Khamis-Roche method for the %PAH). Interpreting findings from research investigations that have used contrasting methods to assess or estimate biological maturity should be done with caution.

Secondly, it is important to note the broader socio-cultural factors that influence selection into, and out of, talent development systems. Naturally, with less female youth participating in organised football relative to males [38], the ‘pool’ to select from at the international level is lower than it is for males. It is generally recognised that increased levels of competition can significantly enhance selection biases associated with early advantages [2], and thus, provides another reasonable factor (beyond biological factors) that may contribute to the lower maturity-associated biases observed in females at the population-level. Indeed, with more males generally participating in football than females [38] and with national/international selection occurring two years earlier for males than females in Ireland, this may contribute to the sex differences we discuss in Ireland in our preceding paragraphs. However, talent identification and development programmes differ notably across contexts, and population trends may not necessarily be the same in alternate Football Associations (e.g., [24]). More broadly, factors including population across regions, invested resources, access to facilities, registered coaches, socio-economic status, talent identification processes and objectives, and contact hours will all inevitably influence selection trends across a given population [24].

It is well established that relative biological maturity and relative age are largely independent [28]. Biological maturation is a biological process, with timing primarily attributable to genetic factors [9]. In contrast, advanced relative age does not infer advanced biological maturity, and the oldest player within an age group can be the least biologically mature, and vice versa [28]. The general trend in football-specific male research has been that, from the onset of puberty, selection biases associated with advanced biological maturity have been larger than those associated with relative age [2, 4, 7, 8]. The same conclusion can inferred from our data, where no RAEs existed. Interestingly, Finnegan et al. [24] found the prevalence of RAEs in club players and those selected for national talent development centres, but not those selected for the junior international team in the United States. In an examination of the squads selected for the U17, U19 and senior Women’s European Championship qualifying campaigns, no RAE was observed at any international level [30]. Yet, in an analysis of U17, U20 and senior Women’s World Cup squads, RAEs were observed for U17 and U19 international players, but not for senior players [29]. These findings, again, contrast to those of Romann and Fuchslocher [39] whereby no RAEs have been observed in any of the youth or senior international teams in Switzerland. Thus, findings in respect to the RAE have been largely inconsistent, highlighting the complex and contextual nature of this phenomenon.

The RAE has been attributed to a multitude of mechanisms, such as a combination of cognitive, emotional, motivational, and social factors [40]. In discussions relating to the RAE in male youth soccer, it has been suggested that the RAE may be attributed to a variety of factors including age-related differences in neuromuscular maturation, behavioural development, experience, and training [7]. More recently, it has been suggested that the RAE is a more complex population-level consequence of a constellation of factors that we cannot directly attribute to a defined set of tangible factors, particularly as the extent of proposed advantages (and the factors providing the advantage) differ between individuals and contexts [41]. Although advanced relative age is proposed as providing a direct advantage at the individual level, our data shows the nuance of context; this was not the case from a selection perspective in our data. The contrasting evidence relating to the existence and magnitude of the RAE across contexts highlights that the RAE is a complex contextual phenomenon with many elements still unclear and with no single or obvious mechanism.

We acknowledge that our data is specific to the Irish context for female youth during one international season and caution is advised when extrapolating to alternate contexts. Moreover, non-invasive methods to estimate biological maturity were used, along with a predictive equation based upon American youth of European ancestry [31]. In addition, parental heights were self-reported and subsequently adjusted for overestimation using equations based upon participant samples in the United States [32]. Of particular significance is that we did not conduct such assessments on non-selected players. In this regard, we infer that a bias exists in favour of earlier maturing players in our sample relative to general population values, rather than players who were not selected. In this sense, we cannot ensure that these biases are due to selection per se or alternative factors.

## CONCLUSIONS

Our data demonstrated the existence of biases in favour of early maturing youth female international footballers relative to the general population, which increase with age, although small in magnitude. Moreover, we demonstrate that the RAE does not exist in this sample, illustrating the complex, contextual, and nuanced nature of the RAE. We suggest the need for further research of this nature, specific to female samples, with a focus on younger ages at the preceding stages of the pathway prior to international selection. The relationship between maturity and key performance metrics should also be investigated, along with the broader dynamics associated with both advanced and delayed biological maturation in female youth.
